# Bone Marrow-Derived Macrophage NLRP3 Mediates Renal Fibrosis by triggering TGF-β/Smad3-mediated Macrophage-Myofibroblast Transition

**DOI:** 10.7150/ijbs.131452

**Published:** 2026-04-23

**Authors:** Wenbiao Wang, Jiaxiao Li, Yu Zhong, Junzhe Chen, Liumei Wu, Lin Wan, Xiao-Ru Huang, Zhiming Ye, Xueqing Yu, Hui-Yao Lan

**Affiliations:** 1 Department of Nephrology, Guangdong Provincial People's Hospital (Guangdong Academy of Medical Sciences), Southern Medical University, Guangzhou, China.; 2 Guangdong-Hong Kong Joint Laboratory on Immunological and Genetic Kidney Diseases, Guangzhou, China.; 3 Department of Medicine & Therapeutics, The Chinese University of Hong Kong, Hong Kong, China.; 4 Department of Nephrology, The Third Affiliated Hospital, Southern Medical University, Guangzhou, China

**Keywords:** NLRP3, MMT, TGF-β/Smad3, profibrotic, renal fibrosis

## Abstract

NLRP3 is a well-recognized pro-inflammatory mediator in renal inflammation. Here, we report a new role for NLRP3 in the pathogenesis of renal fibrosis. Using a large-scale single-cell RNA sequencing, we found that NLRP3 is mainly expressed by macrophages, but not by tubular cells. Functionally, we unexpectedly found that NLRP3 is profibrotic, as mice lacking NLRP3 or macrophage-specific deletion of NLRP3 were protected from UUO and ischemia reperfusion injury (IRI)-induced renal fibrosis. Mechanistically, we uncovered that NLRP3 directly bound TGF-β receptors II and I to trigger the activation of TGF-β/Smad3 signaling and progressive renal fibrosis via the macrophage-myofibroblast transition (MMT) process. This was further confirmed by pharmacological inhibition in a mouse model of UUO in which blockade of NLRP3 inhibited TGF-β/Smad3 signaling, MMT, and progressive renal fibrosis. In conclusion, macrophage NLRP3 is profibrotic and mediates renal fibrosis via the TGF-β/Smad3-MMT mechanism. Targeting macrophage NLRP3 may be a promising therapeutic approach for CKD.

## Introduction

NOD-like receptor (NLR) family pyrin domain-containing 3 (NLRP3), one of the host pattern recognition receptors (PRRs), can recognize the pathogen-associated molecular patterns (PAMPs) and danger-associated molecular patterns (DAMPs) to cause inflammatory responses by releasing the inflammatory cytokines IL-1β and IL-18^1^. Increasing evidence reveals that activation of NLRP3 inflammasome is crucial for the host defense against pathogen invasion 2 and plays a pathogenic role in various inflammatory diseases, such as type 2 diabetes and Alzheimer's disease 3, acute kidney injury (AKI) and chronic kidney disease (CKD) including diabetic kidney disease (DKD) 4-6.

Unresolved renal inflammation can ultimately cause renal fibrosis, a final common pathway leading to the end stage of CKD and kidney failure 7. Renal fibrosis is characterized by excessive extracellular matrix (ECM) deposition accompanied by the loss of nephrons, infiltration of macrophages and T cells, and overexpression of a number of inflammatory cytokines and profibrogenic factors 8. Myofibroblast, characterized by expression of alpha-smooth muscle actin (α-SMA), is the principal cell type responsible for collagen production during renal fibrosis 9. Myofibroblasts are heterogeneous and can originate from a number of sources, including epithelial-mesenchymal transition (EMT) 10, endothelial-mesenchymal transition (EndoMT) 11, proliferation of local resident fibroblasts or pericytes 12, and a newly identified macrophage-myofibroblast transition (MMT) 13. Indeed, pro-inflammatory macrophages are largely derived from bone marrow monocytes and are a key innate immune cell responsible for the process of renal inflammation, repair, and fibrosis in human and experimental kidney disease 14. Macrophages become activated and polarized into different phenotypes in response to the local immune microenvironment 15. Our recent studies identified that bone marrow-derived macrophages can become ECM-producing myofibroblasts during renal fibrosis via TGF-β/Smad3-dependent mechanism 13, 16, 17. Indeed, activation of TGF-β/Smad3 plays a driving role in MMT and ECM synthesis during renal fibrosis 18, 19. The intensity of TGF-β/Smad3 signaling is a critical determinant of macrophage polarization, whereas modulation of the TGF-β/Smad3 axis can restrain aberrant MMT 20. Notably, NLRP3 and Smad3 exhibit bidirectional regulation. It is reported that Smad3 can bind to the promoter region of the NLRP3 gene, thereby contributing to renal inflammation in diabetic nephropathy 21. Furthermore, NLRP3 has been shown to participate in pulmonary inflammation and fibrosis 22, immune modulation in tumor cells 23, and oral submucosal fibrosis 24 through the activation of Smad3 signaling.

Although NLRP3 is well known in the pathogenesis of acute renal inflammation 25, it remains unexplored whether NLRP3 is involved in the progression from acute renal inflammation to chronic renal fibrosis. It is also interest to know whether macrophage-specific NLRP3 is important in this process and what mechanisms of NLRP3 regulate macrophage-mediated renal fibrosis. To address these questions, we first integrated and analyzed large-scale single-cell transcriptomic datasets from CKD patients and unilateral ureteral obstruction (UUO) mice to define the NLRP3- expressing cells. We then explored the functional role of NLRP3, particularly macrophage-specific NLRP3, in UUO and ischemia reperfusion injury (IRI)-induced renal fibrosis in NLRP3 knockout (KO) and macrophage-specific NLRP3 CKO mice. Mechanisms of NLRP3 in macrophage-mediated renal fibrosis via MMT were also investigated, and the therapeutic potential for renal fibrosis by targeting NLRP3 was also explored.

## Materials and Methods

Additional details regarding all methods are provided in the Supplementary Materials and Methods.

### UUO and IRI-induced mouse models

Nlrp3-KO mice (Cat. NO. NM-KO-190428), Nlrp3-Flox mice (Cat. NO. NM-CKO-190002) and Lyz2-Cre (Cat. NO. NMX-KI-192007) mice were purchased from Shanghai Model Organisms Center, Inc. C57BL/6 mice were purchased from Guangdong Experimental Animal Center. All mice in this study were male and aged 8-12 weeks.

A UUO model was performed by ligation of the left ureter in mice as previously described 26. Groups of 6 mice were sacrificed at day 7 after UUO. An ischemic mouse model of renal fibrosis was induced by a well-established IRI in mice for 30 min by mouse arterial clamps. Control mice received the sham-operation procedures without clamping the renal arteries. All procedures were performed under 37℃ and mice after surgery received 5% glucose and NaCl intraperitoneally for volume supply and buprenorphine intramuscularly for analgesia. Groups of 6 mice were sacrificed at day 14 after IRI.

To develop a therapeutic strategy for CKD, a new NLRP3 inhibitor, Licochalcone B (LicoB), at a dose of 40 mg/kg/day, was selected as an optimal dosage for the seven-daytreatment of UUO-induced renal fibrosis in groups of 6 mice. All animal experimental protocols were approved by the Animal Ethics Experimentation Committee at Guangdong Provincial People's Hospital (KY2023-990-01).

### Cell clustering of single-cell RNA-seq data

The gene × cell UMI count matrices were used for cell clustering analysis with Seurat (version 4.1.0) 27. Cells of low quality were identified as those having fewer than 200 expressed genes (with UMI > 0) or exhibiting more than 25% of UMI counts derived from mitochondrial genes, and these were subsequently excluded. Following this, log1p normalization was applied to the gene count matrix. The next step involved identifying the top 3000 highly variable genes using the “FindVariableFeatures” function to facilitate principal component analysis. After that, Louvain clustering was performed alongside Uniform Manifold Approximation and Projection (UMAP) visualization, utilizing 20 principal components at a resolution set to 0.6. A detailed literature review and analysis of known marker expression patterns were then conducted to annotate each cluster.

Processed single-cell RNA sequencing (scRNA-seq) data of fluorescence-activated cell sorting (FACS) sorted CD10 - human CKD samples from Kuppe *et al*. is available from the Zenodo data archive with accession number: 4059315^28^. ScRNA-seq data of 16 kidney allograft samples from Lamarthée *et al*. is available from the European Nucleotide Archive (ENA) with accession number: PRJEB55286^29^. Processed scRNA-seq data of 3 kidney biopsies from Suryawanshi *et al*. is available from the GEO with accession number: GSE151671^30^. ScRNA-seq data of UUO mouse model from Conway *et al*. is available from the GEO with accession number: GSE145053 31.

### Cell lines and cell culture

Human embryonic kidney (HEK293T), human tubular epithelial cells (TECs, HK-2), mouse TECs (TCMK1) and human acute monocytic leukemia cells (THP-1) were purchased from the American Type Culture Collection (ATCC) (Manassas, VA, USA). Primary mouse TECs were separated from C57BL/6 mice kidney, while the mTEC line was a gift from Dr. Jeffrey B.Kopp (NIH). Mouse BMDMs were differentiated from fresh bone marrow cells of NLRP3 WT mice and NLRP3 KO mice in RPMI-1640 medium containing 10% heat-inactivated fetal bovine serum (FBS) in the presence of M-CSF (50 ng/mL, Peprotech 315-02) for 7 days. THP-1 NLRP3 KO cells were constructed by Ubigene Biosciences. THP-1 cells and BMDMs were cultured in RPMI 1640 medium (Gibco, Grand Island, NY, USA) supplemented with 10% heat-inactivated FBS, 100 U/ml penicillin, and 100 μg/ml streptomycin sulfate. HEK293T and TCMK1 cells were cultured in Dulbecco's modified Eagle's medium (DMEM) (Gibco, Grand Island, NY, USA) supplemented with 10% FBS, 100 U/ml penicillin, and 100 μg/ml streptomycin sulfate.HK-2, primary mouse TEC and mTEC cells were cultured in DMEM/F12 medium (Gibco, Grand Island, NY, USA) supplemented with 10% FBS, 100 U/mL penicillin, and 100 μg/mL streptomycin sulfate. Cells were stimulated with or without TGF-β1(5 ng/mL, Peprotech 100-21C) for different time points. All cells were maintained in an incubator at 37°C in a humidified atmosphere of 5% CO_2_.

### Renal histopathology

Kidney tissues were fixed with Histochoice Tissue Fixation MB (AMRESCO, VWR Life Science, PA, USA). PAS staining (Solarbio, G1281) and Masson's trichrome staining (Solarbio, G1340) were performed in paraffin sections (3 μm). The fibrotic area was counted in six random areas of kidney sections in each mouse by the Image-Pro Plus 6.5 quantitative image analysis system (Media Cybernetics, Rockland, MD, USA) and results were expressed as a percentage of the examined area.

### Immunohistochemistry

Immunohistochemistry was performed on paraffin-embedded tissue sections using endogenous horseradish peroxidase blocking and heat-induced epitope retrieval in citrate buffer method 26. The primary antibodies used in this study included p-Smad3 (Rockland 600-401-919), α-SMA (Abcam 230458), and collagen I (Southern Biotech 1310-01). Positive signals were quantitatively analyzed by the Image-Pro Plus 6.5 quantitative image analysis system (Media Cybernetics, Rockland, MD, USA) as previously described 32.

### Two-color flow cytometry analysis

Single-cell suspensions were prepared from kidneys of UUO mice, then digested with Blendzyme 4 (Roche) and fixed by IC Fixation Buffer (eBioscience) for 30 min. Cells were then stained with phycoerythrin (PE)-conjugated mouse CD68 antibody (Ebioscience 12-0689-42) and FITC-conjugated with α-SMA (Sigma F3777) that was conjugated with Pacific Blue by Lighting-Link Fluorescein kit (Invitrogen P30013). After being washed with phosphate-buffered saline (PBS), the stained cells were measured by FACS Caibur flow cytometer (BD Biosciences). All data were analyzed by FlowJo software (v.10.8.1, TreeStar, Ashland, OR, USA).

### Biolayer interferometry assays (BLI)

TGF-β receptor II protein was biotinylated and purchased from MCE (HY-P78215). NLRP3 (LRR) protein with his-tag was purchased from Feiyue Biotechnology (FY-P526451). LicoB was purchased from Selleck (E0225). The buffer and protein diluent used in the experiment was 0.02% Tween 20/PBS buffer. BLI measurements were performed on an Octet R8 instrument (Sartorius).

For the experiment of NLRP3 (LRR) - LicoB interaction, the NLRP3 (LRR) protein was immobilized on a His sensor. LicoB was diluted with PBS containing 1% DMSO and 0.02% Tween 20. The control group was detected by sensors in buffer.

For the experiment of TGF-β receptor II-NLRP3 (LRR) interaction, TGF-β receptor II protein was immobilized on streptavidin sensors. Quenching was carried out with 10 μg/mL biocytin in PBS. After testing the baseline, the sensor tested different concentrations of NLRP3 (LRR) protein. The control group was detected by sensors in protein-free buffer.

For the experiment in which LicoB interfered with TGF-β receptor II-NLRP3 (LRR) interaction, TGF-β receptor II protein was immobilized on streptavidin sensors. Different concentrations of LicoB were diluted using 1% DMSO and 0.02% Tween 20 in PBS. Various concentrations of LicoB were incubated with NLRP3 (LRR) protein for 1 h at room temperature prior to the start of the experiment, followed by BLI.

### Statistical analysis

Each experiment was repeated at least three times. All values were expressed as the mean ± SEM. One-way ANOVA with Turkey's test was used when comparing one factor among multiple groups. Two-way ANOVA with Turkey's test was performed when different parameters between 2 genotypes were compared (GraphPad Software, San Diego, CA, USA) for multiple groups. P values less than 0.05 were considered statistically significant.

## Results

### Single-cell RNA-seq reveals that *NLRP3* is mainly expressed by myeloid cells, presumably macrophages, but not by renal tubular cells in the fibrotic kidney in CKD patients and UUO mice

**Figure 1:**
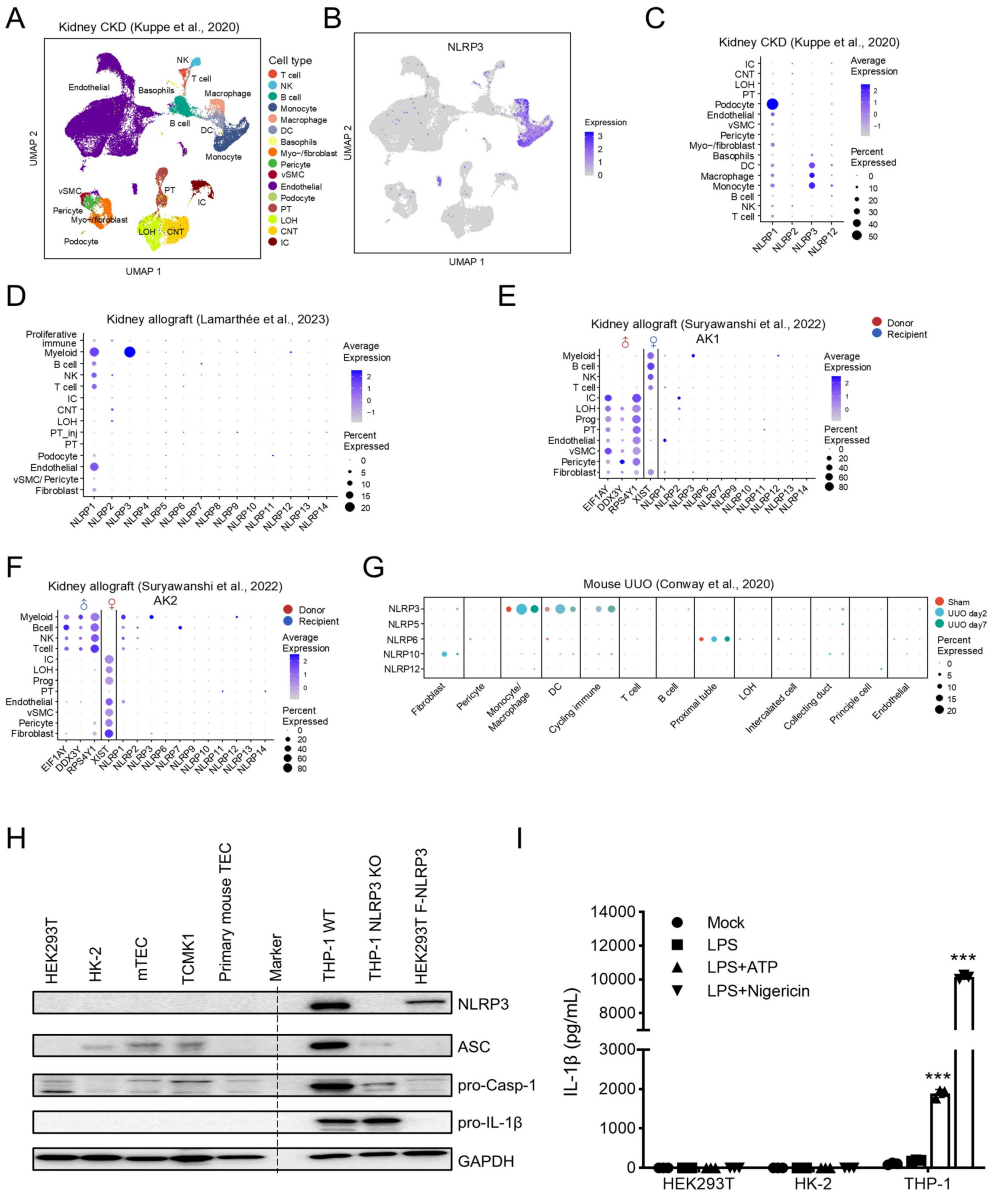
** Single-cell RNA-seq reveals that *NLRP3* is mainly expressed in myeloid lineage like monocytes, dendritic cells and macrophages.** (A) UMAP shows the cell clusters of human fibrotic kidney from CKD patients. Cell types in each dataset are annotated according to representative markers. (B) UMAP shows cell expression of *NLRP3.* (C) Bubble plot shows the expression of *NLRP1*, *NLRP2*, *NLRP3* and *NLRP12* in each cell type from CKD kidneys. (D) Bubble plot shows the expression of NLR family genes in each cell type in allograft kidneys. (E and F) Bubble plot shows the expression of the female X chromosome (*XIST*), male Y chromosome (*RPS4Y1*, *EIF1AY*, and *DDX3Y*), and NLR family genes in each cell type in two allograft kidneys which are recipient-donor sex mismatch. (G) Bubble plot shows the expression of NLR family genes in each cell type in unilateral ureteral obstruction (UUO) mouse model at day 2 and day 7. Bubble size indicates the proportion of cells of inferred cell type expressing each marker; color intensity represents the average expression level. vSMC, vascular smooth muscle cells; NK, natural killer cells; PT, proximal tubular cells; IC, intercalated cells; LOH, loop of Henle; CNT, connecting tubule. (H)Western blotting for the expression of NLRP3 inflammasome components in kidney tubular epithelial cells and TPA-differentiated THP-1 macrophages. Human TECs (HEK293T and HK-2). Mouse TECs (mTEC and TCMK1). The primary mouse TECs were separated from C57BL/6 mice kidneys. HEK293T cells are transfected with pFlag-NLRP3 as a positive control. GAPDH is used as the loading control. (I) HEK293T, HK-2 and TPA-differentiated THP-1 macrophages are stimulated by LPS (1 μg/ml) for 6 h, 5 mM ATP for 2 h or 2 μM Nigericin for 30 min. IL-1β levels in the supernatants are determined by ELISA. ***p < 0.001 versus Mock group.

ScRNA-seq is a new method to determine the cell type-specific expression patterns in distinct cell populations. To systematically investigate the expression of *NLRP3*, we first analyzed publicly available scRNA-seq datasets of human fibrotic kidney from CKD patients, and then conducted cell clustering to identify distinct cell populations in the kidney, including renal intrinsic cells and bone marrow-derived cells (Figure 1A) 28. We found that *NLRP3* was mainly expressed by myeloid-lineage cells (~92%) including monocytes/macrophages and dendritic cells, but not by renal tubular cells, in the CKD kidney (Figure 1B and C; Supplementary Table 1). Data from another study with kidney allograft rejection also confirmed this finding (Figure 1D) 29. To determine whether *NLRP3* is derived from tissue-resident or bone marrow-derived cells, we analyzed a dataset of human kidney allograft biopsies by the expression of X and Y chromosome-specific genes from recipient-donor sex-mismatched kidney transplantation and found that NLRP3-expressing cells originated from the recipient's myeloid cells (Figure 1E; Figure 1F) 30. Similarly, *NLRP3* was also primarily expressed by myeloid cells, but not by renal tubular cells, in a mouse model of UUO (Figure 1G) 31. To further validate these findings, we examined the expression of NLRP3 inflammasome components *in vitro* in well-characterized kidney TEC lines and macrophages by western blotting and found that NLRP3 was highly expressed in macrophages but not in kidney TECs (Figure 1H). Further studies also showed that the addition of NLRP3 specific activators (ATP and Nigericin) was capable of significantly inducing the secretion of IL-1β from macrophages but failed in human TECs (HK-2 and HEK293T) (Figure 1I). Taken together, these findings demonstrated that *NLRP3* was mainly expressed by myeloid cells, specifically by macrophages, but not by TECs in human and mice.

### NLRP3 is profibrotic and deletion of NLRP3 or macrophage-specific NLRP3 protects against renal fibrosis induced in UUO and IRI mice

**Figure 2:**
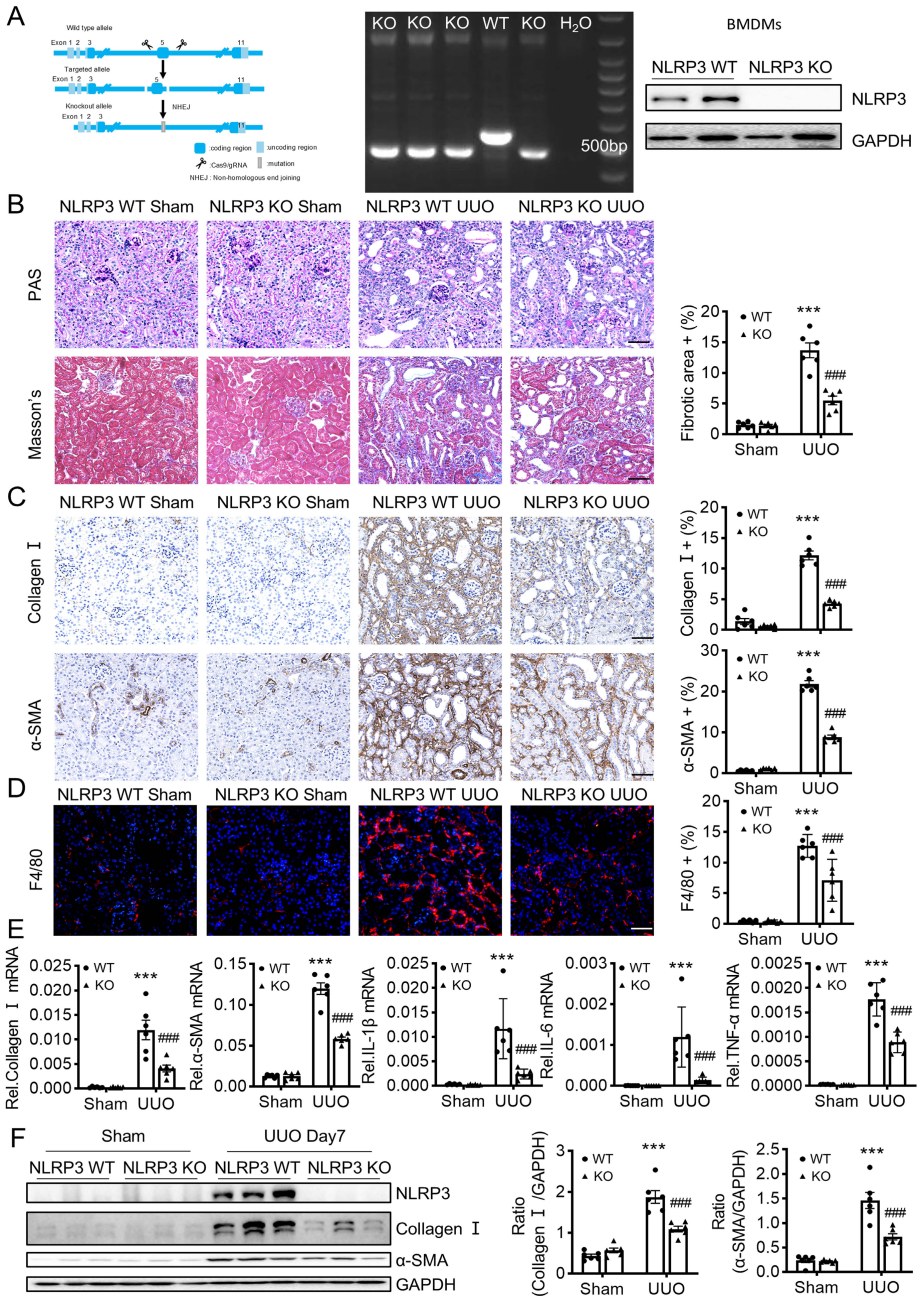
** Mice lacking NLRP3 are protected from UUO-induced renal fibrosis.** (A) NLRP3 KO mice are constructed by CRISPR/Cas9 technology(left). Genotyping results are detected by DNA gel electrophoresis (middle). Western blotting for representative expression of NLRP3 in bone-marrow-derived macrophages (BMDMs) (right). Construct the renal fibrosis mouse model by UUO in NLRP3 WT and NLRP3 KO mice, mice were sacrificed at day 7 post-UUO. (B) Renal fibrosis determined by PAS staining and Masson's trichrome staining (left). Semiquantitative analysis of the fibrotic area in Masson's trichrome staining (right). (C) Immunohistochemistry for detecting collagen I and α-SMA. (D) Immunofluorescence for detecting F4/80. (E) Real-time PCR for levels of collagen I, α-SMA, IL-1β, IL-6 and TNF-α. (F) Western blotting for expression of NLRP3, collagen I and α-SMA on day 7 UUO. Each dot represents one mouse. Data are expressed as the mean ± SEM for groups of six mice. ***p < 0.001 versus NLRP3 WT Sham; ###p < 0.001 versus NLRP3 WT UUO Day 7. Scale bars = 50 μm.

**Figure 3:**
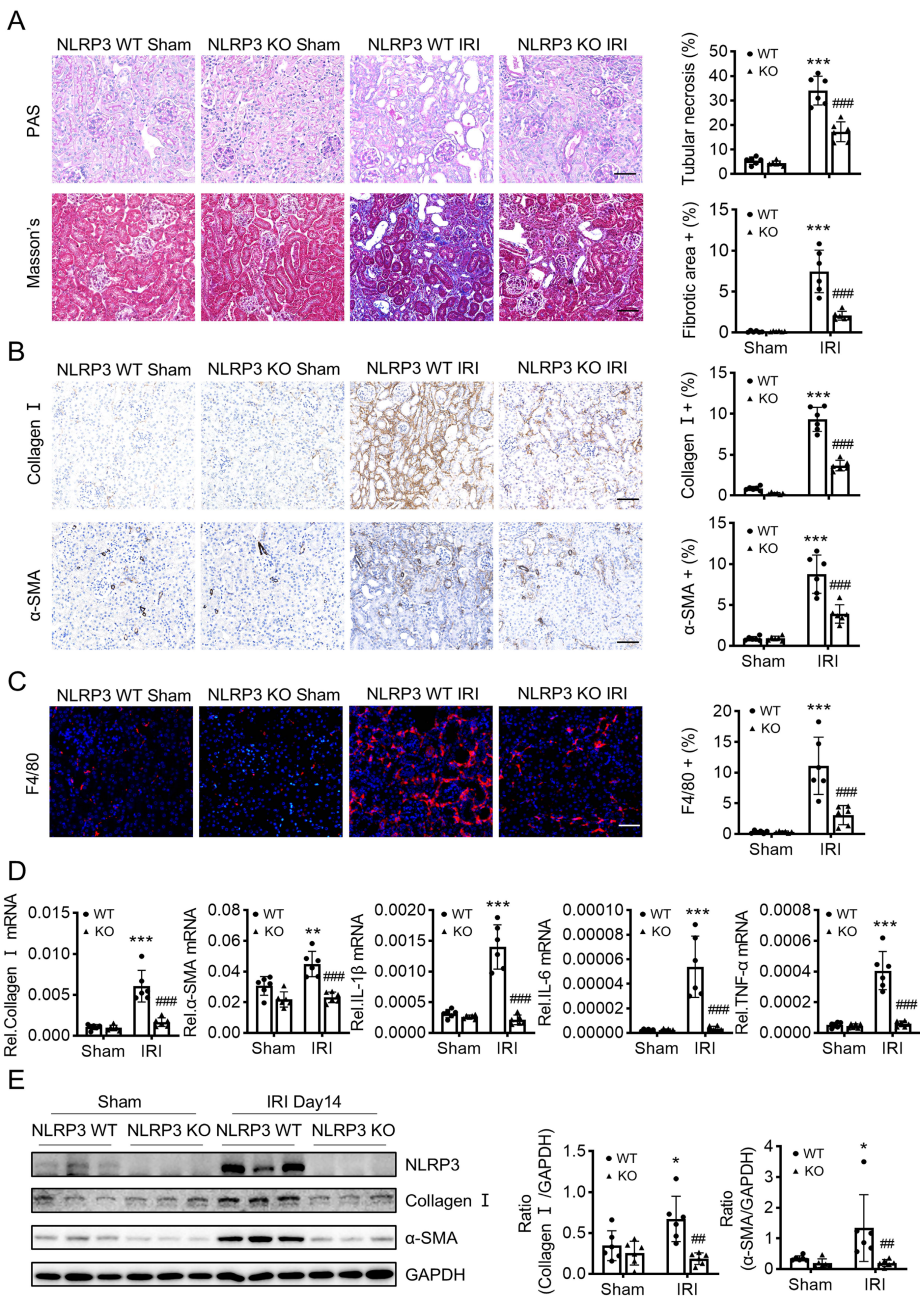
** Mice lacking NLRP3 are protected from IRI-induced renal fibrosis.** Construct the renal fibrosis mouse model by a 30-min ischemia reperfusion injury (IRI) in NLRP3 WT and NLRP3 KO mice, and mice were sacrificed at day 14 post-IRI. (A) Renal fibrosis determined by PAS staining and Masson's trichrome staining (left). Semiquantitative analysis of the fibrotic area in Masson's trichrome staining (right). (B) Immunohistochemistry for detecting collagen I and α-SMA. (C) Immunofluorescence for detecting F4/80. (D) Real-time PCR for levels of collagen I, α-SMA, IL-1β, IL-6 and TNF-α. (E) Western blotting for expression of NLRP3, collagen I and α-SMA. Each dot represents one mouse. Data are expressed as the mean ± SEM for groups of six mice. *p < 0.05, **p < 0.01, ***p < 0.001 versus NLRP3 WT Sham; ##p < 0.01, ###p < 0.001 versus NLRP3 WT IRI. Scale bars = 50 μm.

UUO is a classic model to study CKD caused by progressive tubulointerstitial fibrosis 33. NLRP3 KO mice was reported to reduce renal inflammation and fibrosis in UUO 4. By contrast, another study demonstrated that no change in renal inflammation or fibrosis in NLRP3 KO compared with wild-type mice in UUO 34. To confirm the role of NLRP3 in UUO-induced renal fibrosis, we first constructed the NLRP3 KO mice (Figure 2A) and found that mice lacking NLRP3 were protected from UUO-induced renal fibrosis as demonstrated by renal histopathology (PAS staining and Masson's staining) (Figure 2B), collagen I and α-SMA mRNA and protein expression by real-time PCR, immunohistochemistry, and western blot analysis (Figure 2C, E-F). Furthermore, we investigated the role of NLRP3 on the inflammatory response in the UUO mouse model via immunofluorescence and qPCR. The results showed that knockout of NLRP3 significantly inhibited macrophage infiltration and the mRNA expression levels of inflammatory cytokines, including IL-1β, TNF-α, and IL-6 (Figure 2D-E). We then examined the role of NLRP3 in another model of renal fibrosis, induced by IRI, which is a well-established mouse model to explore AKI-induced renal fibrosis 35. Similar to those findings in the UUO kidney, deletion of NLRP3 also prevented IRI-induced renal inflammation and fibrosis (Figure 3).

**Figure 4:**
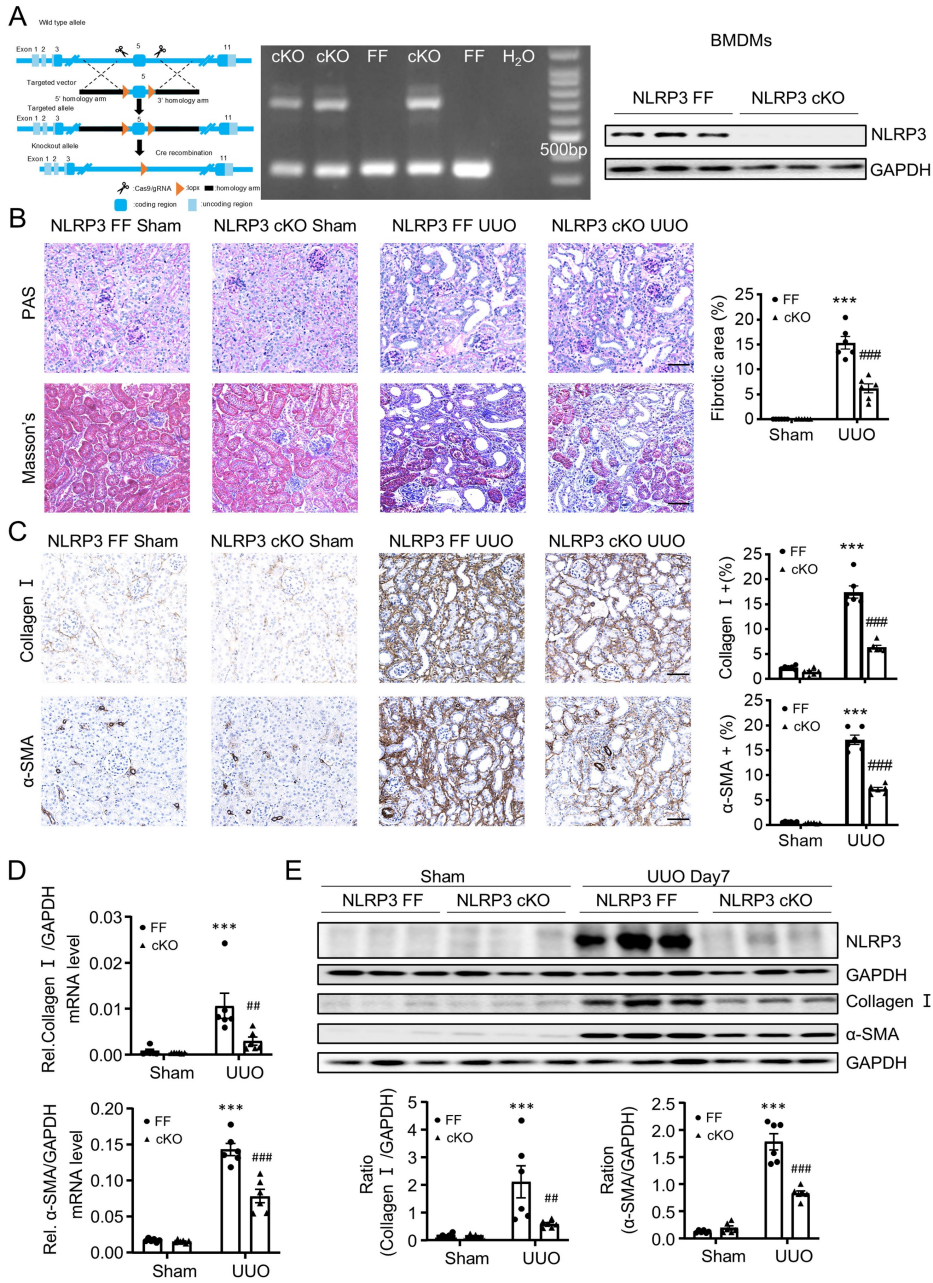
** Macrophage-specific NLRP3 deficiency prevents from UUO-induced renal fibrosis in mice.** (A) Construct the NLRP3 flox/flox mice by CRISPR/Cas9 and in-fusion cloning technology and then mating with Lyz2-Cre mice to get the macrophage-specific NLRP3 deficiency offspring mice (left). Genotyping results are detected by DNA gel electrophoresis (middle). Western blotting for representative expression of NLRP3 in BMDMs (right). Construct the renal fibrosis mouse model by UUO in NLRP3 flox/flox and NLRP3 flox/flox Lyz2-Cre mice, mice were sacrificed at day 7 post-UUO. (B) Renal fibrosis determined by PAS staining and Masson's trichrome staining(left). Semiquantitative analysis of fibrotic area in Masson's trichrome staining(right). (C) Immunohistochemistry for detecting collagen I and α-SMA. (D) Real-time PCR for levels of collagen I and α-SMA. (E) Western blotting for expression of NLRP3, collagen I and α-SMA on day7 UUO. Each dot represents one mouse and data are expressed as the mean ± SEM for groups of six mice. ***p < 0.001 versus NLRP3 Flox/Flox Sham; ##p < 0.05, ###p < 0.001 versus NLRP3 Flox/Flox UUO Day7. Scale bars = 50 μm.

**Figure 5:**
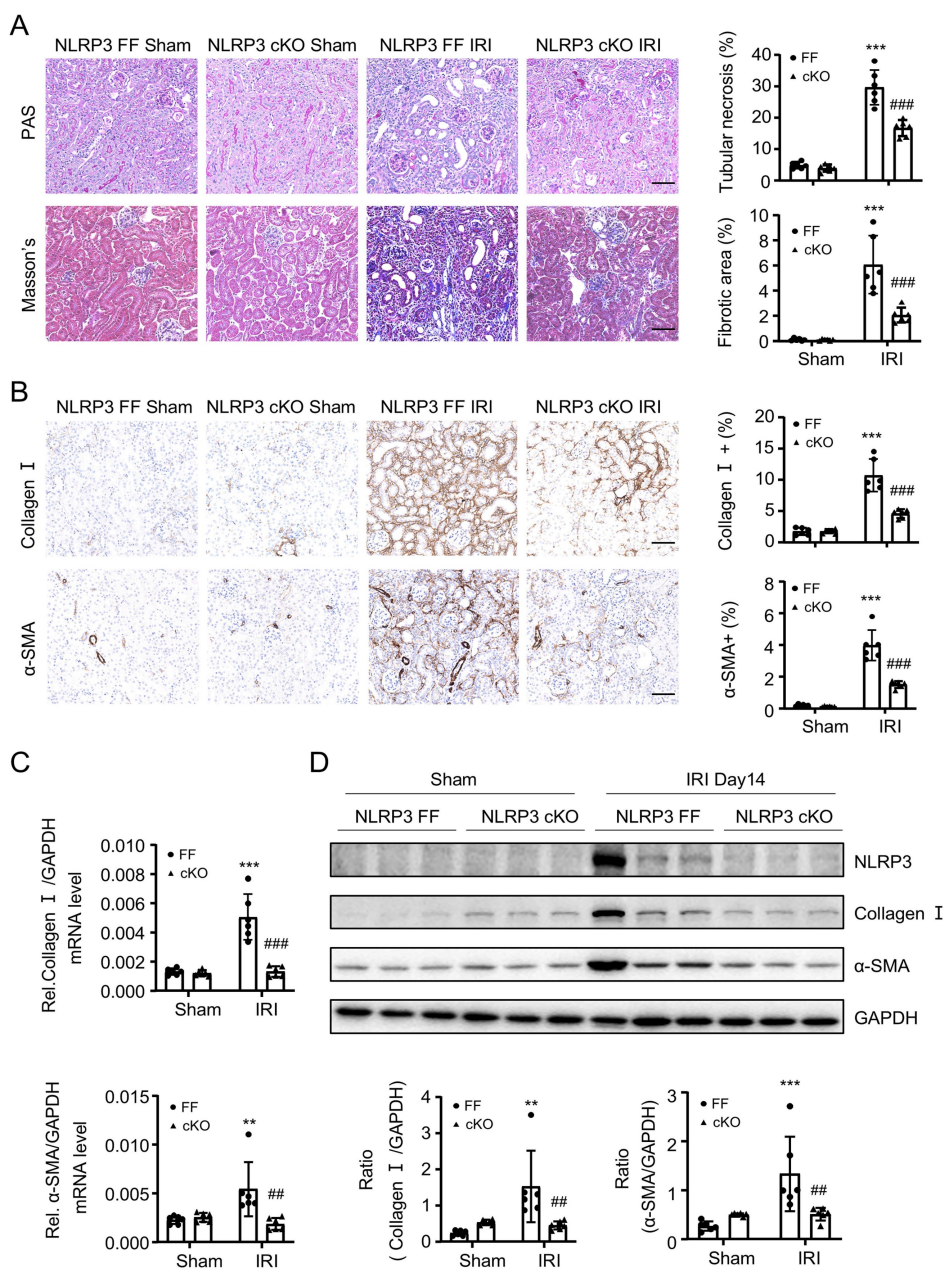
** Macrophage-specific NLRP3 deficiency prevents from IRI-induced renal fibrosis in mice.** Construct the renal fibrosis mouse model by a 30-min IRI in NLRP3 flox/flox and NLRP3 flox/flox Lyz2-Cre mice, and mice were sacrificed at day 14 post-IRI. (A) Renal fibrosis determined by PAS staining and Masson's trichrome staining (left). Semiquantitative analysis of fibrotic area in Masson's trichrome staining (right). (B) Immunohistochemistry for detecting collagen I and α-SMA. (C) Real-time PCR for levels of collagen I and α-SMA. (D) Western blotting for expression of NLRP3, collagen I and α-SMA. Each dot represents one mouse and data are expressed as the mean ± SEM for groups of six mice. **p < 0.01, ***p < 0.001 versus NLRP3 Flox/Flox Sham; ##p < 0.01, ###p < 0.001 versus NLRP3 Flox/Flox IRI. Scale bars = 50 μm.

Because NLRP3 is highly expressed by macrophages, as demonstrated by scRNA-seq in this (Figure 1A) and other study 36, and macrophages play a critical role in renal fibrosis 14, we next investigated the specific role of macrophage NLRP3 in renal fibrosis by generating macrophage-specific NLRP3 KO mice in which NLRP3 was specifically deleted from macrophages (Figure 4A). Like the global NLRP3 KO mice, deletion of macrophage-specific NLRP3 also equally inhibited both UUO and IRI-induced renal fibrosis (Figure 4B-E and Figure 5). These findings reveal that macrophage-derived NLRP3 may play a key role in UUO and IRI-induced renal fibrosis.

### NLRP3 mediates renal fibrosis by triggering MMT via the TGF-β/Smad3-dependent mechanism *in vivo* and *in vitro*

**Figure 6:**
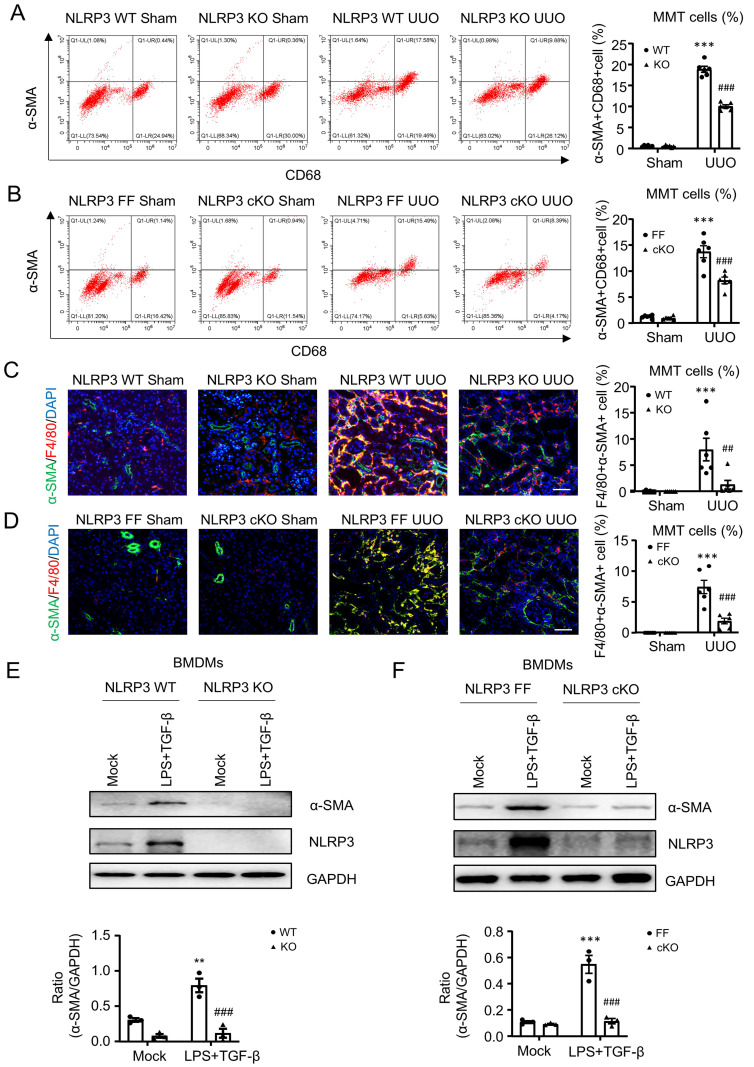
** Macrophage-specific NLRP3 deficiency inhibits MMT in the UUO kidney and TGF-β1-stimulated BMDMs**. (A and B) Flow cytometry analysis of total renal cells derived by enzyme-digested kidney. The plots show double staining for α-SMA with CD68 in NLRP3 KO (A) and NLRP3 CKO (B) on day7 UUO (left). Graphs show the percentage of α-SMA+ and CD68+ in each group (right). (C and D) Tow-color immunofluorescence reveals the expression of MMT cells (α-SMA, green; F4/80, red) in NLRP3 KO (C) and NLRP3 CKO (D) on day7 UUO (left). Graphs show the percentage of Macrophage-Myofibroblast Transition (MMT) cells in each group(right). Each dot represents one mouse and data are expressed as the mean ± SEM for groups of six mice. (E and F) Western blotting for the expression of α-SMA and NLRP3 in BMDMs which are stimulated by LPS (1 μg/mL) for 6 h and TGF-β1 (5 ng/mL) for 24h from NLRP3 KO (E) and NLRP3 CKO (F) mice (Top). Graphs show the expression as means ± SEM for at least three independent experiments. **p < 0.01, ***p < 0.001 versus NLRP3 WT Sham, NLRP3 Flox/Flox Sham, NLRP3 WT Mock or NLRP3 Flox/Flox Mock; ##p < 0.01, ###p < 0.001 versus NLRP3 WT UUO Day7, NLRP3 Flox/Flox UUO Day7, NLRP3 WT LPS+ TGF-β1 or NLRP3 Flox/Flox LPS+ TGF-β1. Scale bars = 50 μm.

As macrophages mediate renal fibrosis via the MMT-dependent mechanism 13, 16, 17, we then examined whether NLRP3 mediates renal fibrosis by triggering the MMT process. Flow cytometry analysis showed that MMT (α-SMA^+^CD68^+^) cells were significantly decreased in NLRP3 KO and macrophage NLRP3 cKO UUO mice (Figure 6A and Figure 6B). This novel observation was further confirmed by two-color immunofluorescence in the fibrosing kidneys induced by UUO and IRI where MMT cells were largely reduced in mice with global or macrophage-specific deletion in UUO mice (Figure 6C, D and Figure S2A, B).

To confirm this *in vivo* finding, we further investigated the pathogenic role of NLRP3 in MMT by using bone-marrow-derived macrophages (BMDMs) under high LPS and TGF-β1 conditions. As expected, the addition of LPS and TGF-β1 largely induced NLRP3 expression and MMT by BMDMs, which was blocked in those lacking NLRP3 (Figure 6E, F). These *in vivo* and *in vitro* findings reveal that NLRP3 mediates renal fibrosis via the MMT-dependent mechanism.

**Figure 7:**
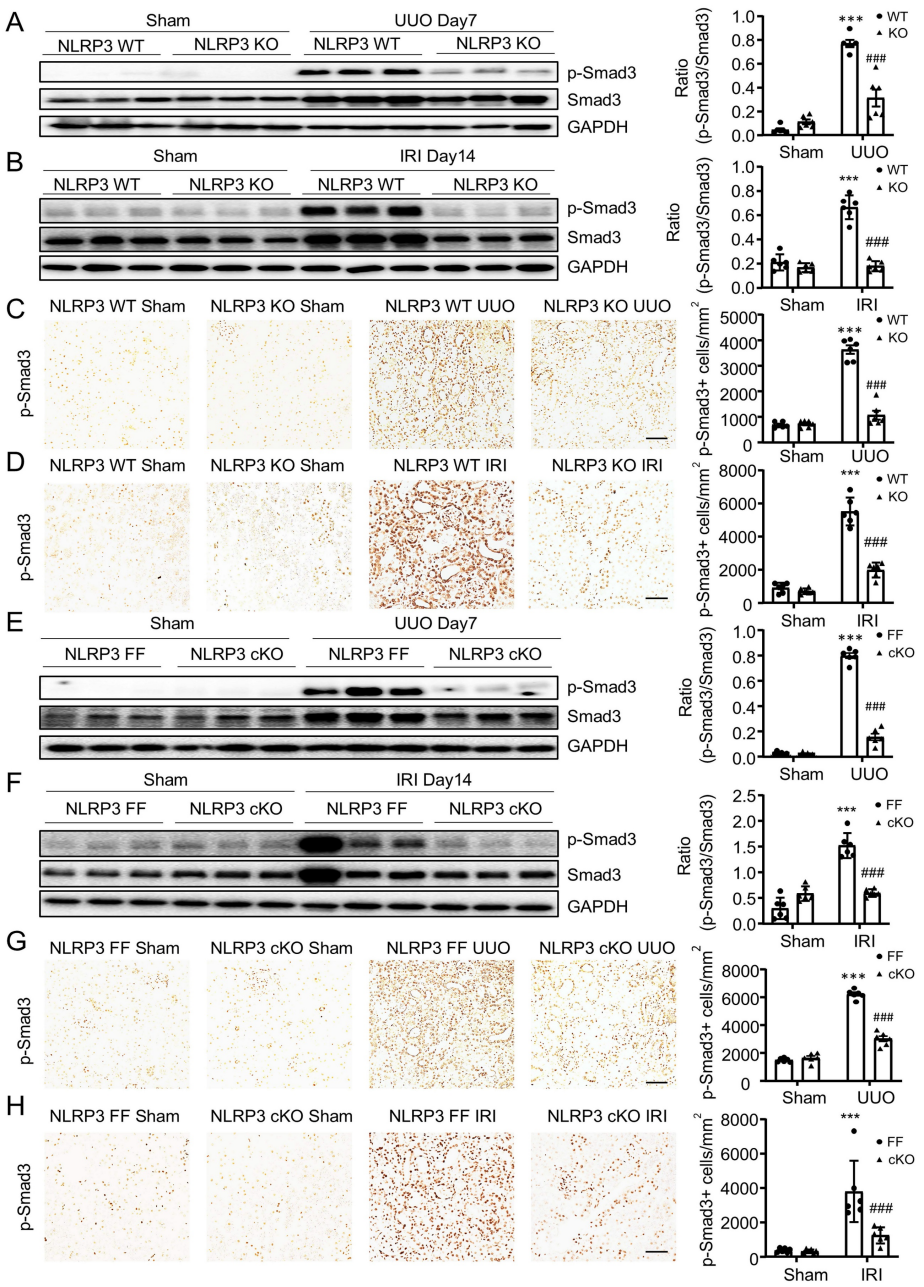
** Macrophage-specific NLRP3 deficiency inhibits TGF-β/Smad3 signaling in UUO kidney and THP-1 macrophages.** (A and B) Western blotting for activation of Smad3 (p-Smad3) and total Smad3 in NLRP3 KO on day 7 UUO(A) and day 14 IRI-induced renal fibrosis (B). (C and D) Immunohistochemistry for detecting activation of Smad3 (p-Smad3) in NLRP3 KO on day 7 UUO(C) and day 14 IRI-induced renal fibrosis (D). (E and F) Western blotting for activation of Smad3(p-Smad3) and total Smad3 in NLRP3 CKO on day 7 UUO (E) and day 14 IRI-induced renal fibrosis (F). (G and H) Immunohistochemistry for detecting activation of Smad3 (p-Smad3) in NLRP3 CKO on day 7 UUO (G) and day 14 IRI-induced renal fibrosis (H). Each dot represents one mouse and data are expressed as the mean ± SEM for groups of six mice. ***p < 0.001 versus NLRP3 WT Sham or NLRP3 Flox/Flox Sham; ###p < 0.001 versus NLRP3 WT UUO Day7, NLRP3 Flox/Flox UUO Day7, NLRP3 WT IRI or NLRP3 Flox/Flox IRI. Scale bars = 50 μm.

It is well established that TGF-β/Smad3 signaling is critical in MMT and renal fibrosis. We then examined whether NLRP3-mediated MMT and renal fibrosis is associated with activation of TGF-β/Smad3 signaling. Western blot and immunohistochemistry analysis showed that deletion of NLRP3 significantly inhibited the activation of Smad3 signaling in the fibrosing kidneys induced by UUO and IRI in NLRP3 KO mice (Figure 7A-D) and macrophage-specific NLRP3 KO mice (Figure 7E-H). These findings reveal that NLRP3 may promote MMT in renal fibrosis via TGF-β/Smad3 signaling.

### NLRP3 interacts directly with the TGF-β1 receptor to trigger the activation of TGF-β/Smad3 signaling

**Figure 8:**
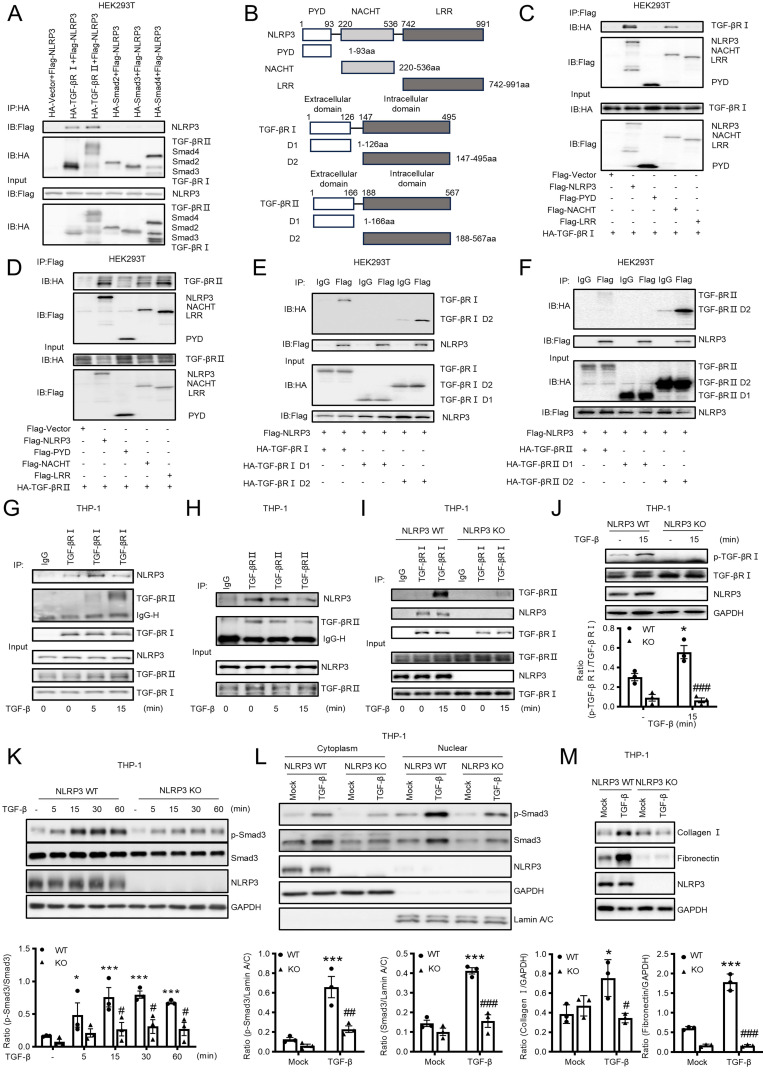
** Co-IP analysis detects the interaction between macrophage NLRP3 and TGF-β receptors I and II.** (A) HEK293T is co-transfected with NLRP3 and TGF-β signaling protein plasmids. (B) The diagrams of NLRP3 and its truncated proteins (PYD, NACHT, and LRR), TGF-β receptor I and its truncated proteins (extracellular and intracellular domain), and TGF-β receptor II and its truncated proteins (extracellular and intracellular domain) (C and D) HEK293T is co-transfected with TGF-β receptor and NLRP3 domains. (E and F) HEK293T is co-transfected with NLRP3 and TGF-β receptor domains, Domain1 (Extracellular domain) and Domain 2 (Intracellular domain). (G and H) TPA-differentiated THP-1 macrophages are stimulated with TGF-β1 (5 ng/mL) for 5min and 15min. (I) TPA-differentiated NLRP3 WT and NLRP3 KO THP-1 macrophages are stimulated with TGF-β1 (5 ng/mL) for 15 min.(A-I) Cell lysates were subjected to Co-IP using antibody and analyzed by immunoblotting using each specific antibody (top) or analyzed directly by immunoblotting using specific antibody (as input) (bottom). (J) THP-1 macrophages are stimulated with TGF-β1 (5 ng/mL) for 15min. Western blotting for the expression of phosphorylation TGF-β receptor I (left). (K) THP-1 macrophages are stimulated with TGF-β1 (5 ng/mL) for each time. Western blotting for activation of Smad3 (p-Smad3), total Smad3 and NLRP3. (L) THP-1 macrophages are stimulated with TGF-β1 (5 ng/mL) for 2 h. Western blotting for the expression of activation of Smad3 (p-Smad3), total Smad3 and NLRP3 in cytoplasmic and nuclear protein fractions. (M) THP-1 macrophages are stimulated with TGF-β1 (5 ng/mL) for 24 h. Western blotting for the expression of Collagen I, Fibronectin and NLRP3. Graphs show the expression as means ± SEM for at least three independent experiments(right). *p < 0.05, ***p < 0.001 versus NLRP3 WT Mock, #p < 0.05, ##p < 0.01, ###p < 0.001 versus NLRP3 WT TGF-β1.

We next examined the mechanism by which NLRP3 activates Smad3 to mediate MMT and renal fibrosis by examining the direct interaction between NLRP3 and components of TGF-β signaling. By using co-immunoprecipitation (Co-IP) with plasmids overexpressing system in HEK293T cells, we clearly identified that NLRP3 could interact directly with TGF-β receptors I and II, but not Smad2, Smad3, and Smad4 (Figure 8A). It is known that the NLRP3 protein harbors several prototypic domains, including the PYRIN domain (PYD), NACHT-associated domain (NAD), and Leucine rich repeats (LRR)^37^. We then determined the domain of NLRP3 involved in the interaction with TGF-β receptor I and II by evaluating the plasmids encoding NLRP3, PYD, NACHT, or LRR as described previously 38. Results revealed that TGF-β receptor I was able to interact with NLRP3 and NACHT, but not with PYD and LRR (Figure 8B-C). TGF-β receptor II also interacted with NLRP3, NACHT and LRR, but not with PYD (Figure 8D). It was noticed that NLRP3 was capable of interacting with the intracellular domain (D2) of TGF-β receptor I and TGF-β receptor II (Figure 8E and Figure 8F). Further studies revealed that there was an endogenous interaction between NLRP3 and the two receptors in TPA-differentiated THP-1 macrophages (Figure 8G and Figure 8H). The interaction between TGF-β receptor I and NLRP3 increased after TGF-β1 stimulation at 5min (Figure 8G), but the interaction between TGF-β receptor II and NLRP3 decreased after TGF-β1 stimulation at 15min (Figure 8H). To explore how NLRP3 modulates TGF-β signaling, we examined the endogenous interaction between NLRP3 and the two TGF-β receptors in NLRP3 KO macrophages and found that the interaction between NLRP3 and TGF-β receptor I and II was significantly decreased in NLRP3 KO macrophages after TGF-β1 stimulation at 15min (Figure 8I). It is well established that TGF-β binds to TGF-β receptor II to recruit and cause phosphorylation of TGF-β receptor I for further signaling activation 39. Then, we examined the function of NLRP3 in the phosphorylation of TGF-β receptor I and showed that the phosphorylation of TGF-β receptor I was significantly decreased in NLRP3 KO macrophages after TGF-β1 stimulation at 15min (Figure 8J). This was also confirmed *in vitro* in a macrophage cell line (THP-1) in which deletion of NLRP3 inhibited TGF-β1-induced Smad3 phosphorylation and nuclear translocation, as well as the expression of collagen I and fibronectin (Figure 8K-M). Results from this study reveal that NLRP3 is required for the activation of TGF-β/Smad3 signaling by interacting with TGF-β receptor I and II.

### Treatment with NLRP3 inhibitor (Licochalcone B) attenuates renal fibrosis by inhibiting TGF-β/Smad3-mediated MMT *in vitro* and in a mouse model of UUO

**Figure 9:**
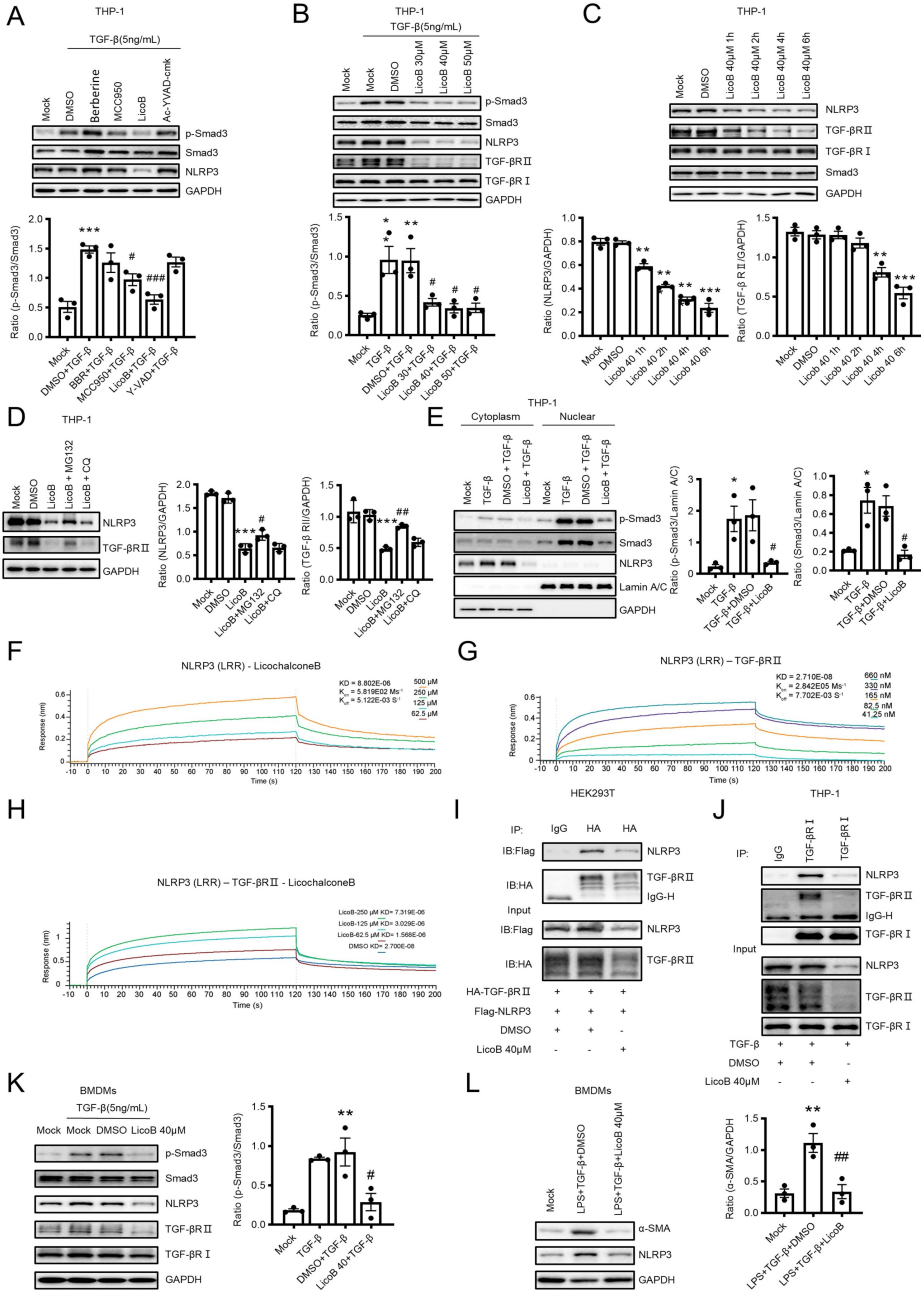
** Treatment with a NLRP3 inhibitor blocks TGF-β/Smad3 signaling and MMT *in vitro*.** (A-C) TPA-differentiated THP-1 macrophages are pre-treated with NLRP3 inflammasome inhibitor Berberine (40μM), MCC950 (10μM), LicoB (40μM), Ac-YVAD-cmk (10μg/mL) for 6 h, and then stimulated with TGF-β1 (5 ng/mL) for 30 min(A). Macrophages are pre-treated with Licob 30, 40, 50 μM for 6 h and then stimulated with TGF-β1 (5 ng/mL) for 30 min (B). Macrophages are treated with Licob 40 for 1 h,2 h,4 h and 6 h (C). Western blotting for the expression of activation of NLRP3, TGF-β receptor I, TGF-β receptor II and Smad3. (D) THP-1 macrophages are pre-treated with MG132 (10 μM), chloroquine (20 μM) for 2 h and then stimulated with Licob (40 μM) for 6 h. Western blotting for the expression of NLRP3 and TGF-β receptor II. (E) THP-1 macrophages are pre-treated with NLRP3 inflammasome inhibitor Licob 40 for 6h and then stimulated with TGF-β1 (5 ng/mL) for 2 h. Western blotting for activation of Smad3 (p-Smad3), total Smad3 and NLRP3 in cytoplasmic and nuclear protein fractions. (F-H) Binding affinity of LicoB or TGF-β receptor II to NLRP3 LRR domain determined by kinetic assays using the Sartorius Octet System. (F)LicoB with NLRP3 LRR domain, (G) TGF-β receptor II with NLRP3 LRR domain, (H) LicoB and NLRP3 LRR domain with TGF-β receptor II. (I) HEK293T is co-transfected with p-HA-TGF-β receptor II and p-Flag-NLRP3 for 24 h and then treated with Licob 40 for 6 h. (J) THP-1 macrophages are treated with Licob 40 for 6 h and then stimulated with TGF-β1 (5 ng/mL) for 15 min. Cell lysates were subjected to Co-IP using antibody and then analyzed by immunoblotting using each specific antibody (top) or analyzed directly by immunoblotting using specific antibody (as input) (bottom). (K) Western blotting for each protein in BMDMs which stimulated by Licob 40 for 6 h and TGF-β1 (5 ng/mL) for 30min. (L) Western blotting for the expression of α-SMA and NLRP3 in BMDMs which were stimulated by Licob 40 for 6 h, LPS (1 μg/ml) for 6 h and TGF-β1 (5 ng/mL) for 24h (up). Data are the means ± SEM for at least three independent experiments. *p < 0.05, **p < 0.01, ***p < 0.001 versus Mock; #p < 0.05, ##p < 0.01, ###p < 0.001 versus TGF-β1+DMSO or LPS+ TGF-β1+DMSO.

We next tested our hypothesis that targeting NLRP3 may be a novel therapy for renal fibrosis. It is reported that Berberine inhibits the NLRP3 inflammasome by decreasing mitochondrial ROS generation 40, whereas MCC950 inhibits the NLRP3 inflammasome by interacting with the NLRP3 NACHT domain 41. LicoB can specifically inhibit the NLRP3 inflammasome by disrupting NEK7-NLRP3 interaction 42. Ac-YVAD-cmk is a specific inhibitor of Caspase-1^43^. We first determined the therapeutic efficacy of these inhibitors in TGF-β-stimulated macrophages *in vitro* and found that the phosphorylation of Smad3 was slightly decreased after treatment with MCC950 but was significantly decreased after treatment with LicoB (Figure 9A). We also observed that LicoB treatment dose-dependently inhibited the expression of NLRP3 by macrophages (Figure 9B). Interestingly, treatment with LicoB significantly inhibited TGF-β1-induced p-Smad3, NLRP3, and TGF-β receptor II without altering the expression of Smad3 and TGF-β receptor I (Figure 9B). Further studies revealed that treatment with LicoB significantly inhibited expression of NLRP3 at 1h and then expression of TGF-β receptor II at 4h without altering TGF-β receptor I and Smad3 (Figure 9C, Figure S3). All these results indicated that LicoB may function to inhibit the interaction between NLRP3 and TGF-β receptor II. We next determined how LicoB regulates NLRP3 and TGF-β receptor II protein expression by adding a proteasome inhibitor, MG132, and found that addition of MG132 significantly attenuated the LicoB-induced downregulation of TGF-β receptor II and NLRP3 protein levels, which was not altered by a lysosomal inhibitor, chloroquine (Figure 9D). These results suggest that LicoB primarily promotes their degradation via the proteasomal pathway. We also demonstrated that treatment with LicoB significantly decreased TGF-β-induced Smad3 nuclear translocation in macrophages (Figure 9E). We then explored the binding affinity of LicoB with the NLRP3 LRR domain by the equilibrium dissociation constant (KD) at 8.8 μM (Figure 9F). Our results showed that the binding affinity of NLRP3 LRR domain with TGF-β receptor II was 27 nM (Figure 9G), which was markedly reduced by incubating with LicoB (62.5 μM,125 μM,250 μM) from 27 nM to 1.6 μM, 3 μM or 7.3 μM (Figure 9H). Thus, treatment with LicoB inhibited the interaction between NLRP3 and TGF-β receptor II in HEK293T cells and in macrophages (Figure 9I and Figure 9J), thereby blocking TGF-β1-induced Smad3 phosphorylation and MMT in BMDMs (Figure 9K and Figure 9L).

**Figure 10:**
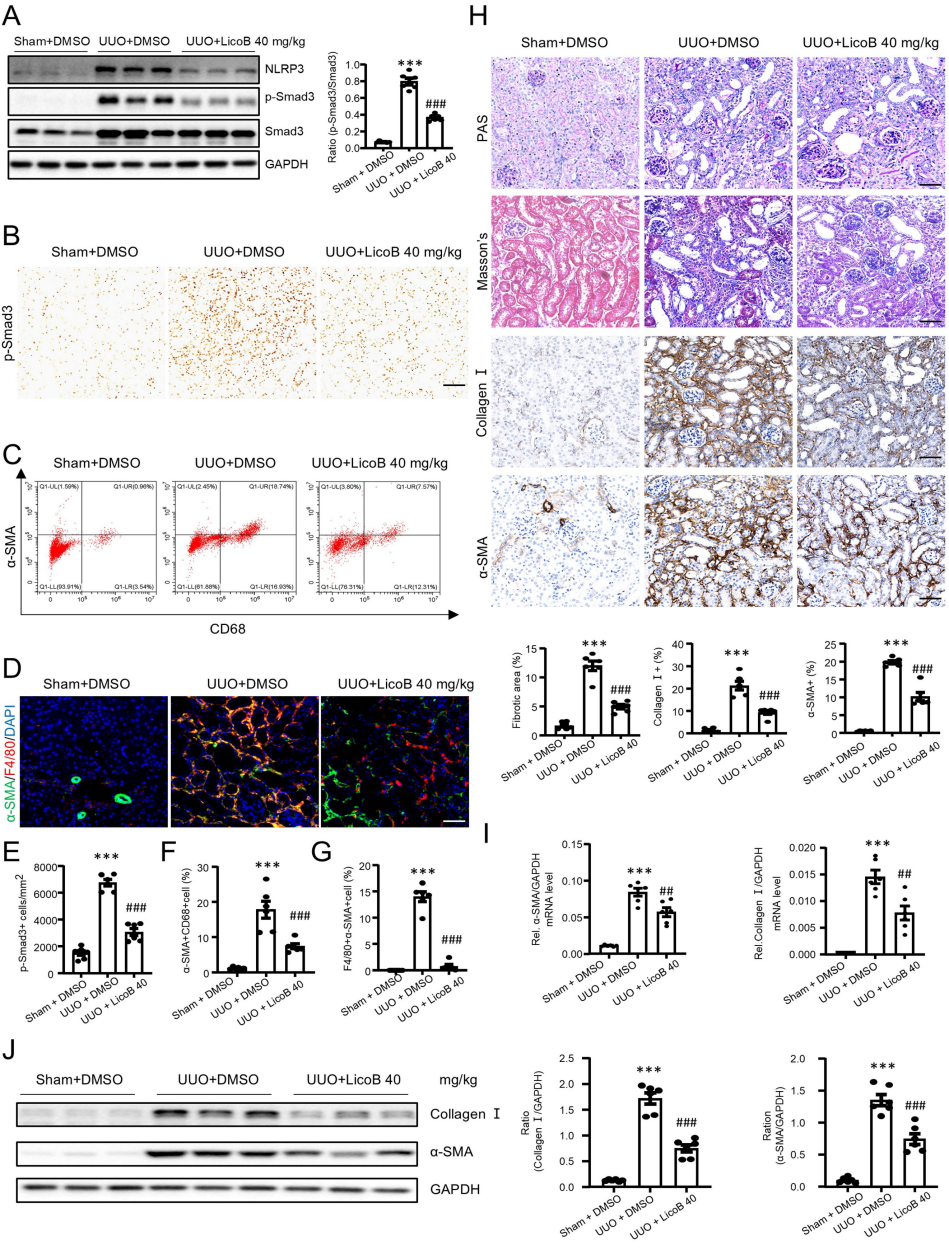
** Treatment with a NLRP3 inhibitor inhibits renal fibrosis by blocking TGF-β/Smad3 signaling and MMT in a mouse model of UUO.** Licochalcone B at a dose of 40 mg/kg/day was treated in UUO. (A) Western blotting for expression of NLRP3, activation of Smad3 (p-Smad3) and total Smad3 on day 7 UUO. (B and E) Immunohistochemistry for detecting activation of Smad3 (p-Smad3) on day 7 UUO (B). Graphs show the numbers of p-Smad3+ cells/mm² in each group (E). (C and F) Flow cytometry analysis of kidney cells derived by enzyme digested kidneys. The plots show double staining for α-SMA with CD68 on day7 UUO (C). Graphs show the percentage of α-SMA+ and CD68+ in each group (F). (D and G) Tow-color immunofluorescence reveals the expression of MMT cells (α-SMA, green; F4/80, red) on day7 UUO(D). Graphs show the percentage of α-SMA+ and F4/80+ in each group (G). (H) PAS staining and Masson's trichrome staining (top). Immunohistochemistry for detecting collagen I and α-SMA (middle). Semiquantitative analysis of fibrotic area in each group(bottom). (I) Real-time PCR for levels of collagen I and α-SMA. (J) Western blotting for expression of collagen I and α-SMA on day7 UUO. Each dot represents one mouse and data are expressed as the mean ± SEM for groups of six mice. ***p < 0.001 versus Sham+DMSO; ##p < 0.01, ###p < 0.001 versus UUO+DMSO. Scale bars = 50 μm.

We next examined whether LicoB can block TGF-β/Smad3 signaling and MMT in a mouse model of UUO by a daily intraperitoneal (i.p.) injection with different doses of LicoB (n=3). We found that all four dosages of LicoB did not cause systemic and other organ toxicities as determined by LDH, ALT, and AST (Figure S4, Supporting Information), but dose-dependently inhibited UUO-induced renal fibrosis, with the best therapeutic efficacy at 40mg/kg as determined by renal histopathology (PAS staining), immunohistochemistry, and real-time PCR for collagen I and α-SMA expression (Figure S5, Supporting Information). Western blot and immunohistochemical analysis showed that LicoB treatment significantly attenuated NLRP3 expression and p-Smad3 in the UUO kidney, with the best inhibitory effect at 40mg/kg (Figure 10A and Figure 10B,10E). Flow cytometry analysis of enzyme-digested kidney tissue and two-color immunofluorescence analysis also revealed that blockade of NLRP3 with LicoB inhibited MMT by largely reducing F4/80+α-SMA+ cells in the UUO kidney, again with the best inhibitory effect at the dose of 40mg/kg (Figure 10C,10F and Figure 10D, 10G). We then found that treatment with LicoB at 40mg/kg attenuated UUO-induced renal fibrosis as demonstrated by renal histopathology (PAS staining and Masson's staining), collagen I and α-SMA mRNA and protein expression by real-time PCR, immunohistochemistry, and western blot analysis (Figure 10H-J). Taken together, treatment with an optimal dose of LicoB (40mg/kg) inhibited expression of NLRP3 and blocked activation of TGF-β/Smad3 signaling, resulting in attenuating MMT and progressive renal fibrosis in the UUO kidney.

## Discussion

In this study, we found that NLRP3 is primarily expressed by macrophages, but not by intrinsic kidney cells such as tubular cells as determined by scRNA-seq. Importantly, we discovered that NLRP3 is profibrotic and macrophage-specific NLRP3 mediates renal fibrosis via a mechanism associated with MMT. This was supported by the findings that mice lacking NLRP3 or macrophage-specific NLRP3 were protected from MMT and progressive renal fibrosis in mouse models of UUO and IRI, and in TGF-β1-stimulated BMDMs. Mechanistically, we uncovered that NLRP3 mediates renal fibrosis by directly interacting with TGF-β1 receptors to promote activation of Smad3 signaling and the MMT process. Thus, targeting NLRP3 with an inhibitor, LicoB, can block MMT and renal fibrosis.

The identification of NLRP3-expressing cells is the first step to explore the function of this protein in kidney disease. Many studies find that NLRP3 is expressed by kidney tubular epithelial cells by immunofluorescence and western blotting 44-46. Recently, Sophie *et al*. showed the expression of NLRP3 in myeloid cells and the absence of NLRP3 transcripts in epithelial cells of the human kidney via single-cell transcriptome analysis 36. RNA-seq data (GEO: GSE88982) also demonstrate that the expression of NLRP3 occurs during hematopoietic stem cell (HSCs) differentiation through the myeloid lineage 47. In the present study, by using scRNA-seq, we found that NLRP3 was mainly expressed by myeloid cells, primarily by macrophages, but absent in renal epithelial cells in the fibrotic kidney of patients and mice with CKD. *In vitro* studies using cell lines also confirmed that NLRP3 was highly expressed by THP-1 macrophages, but not by renal tubular epithelial cells (HEK293T and HK-2). However, due to the limited sensitivity of scRNA-seq, it remains possible that TECs may express very low levels of NLRP3 undetectable by scRNA-seq, or that its expression is fully captured in our model under specific pathological conditions. Our results using sex-mismatched renal allograft rejection further revealed that NLRP3-expressing macrophages were from the recipients, not locally from the donor kidney, suggesting that the NLRP3-expressing cells within the diseased kidney are from circulation. These findings suggest that myeloid NLRP3 may play a key role in kidney disease.

It is well established that NLRP3 is a pro-inflammatory mediator in renal inflammation 48. Focused on the activation of the NLRP3 inflammasome, numerous studies have revealed its pivotal role in mediating inflammatory responses during the early stages of renal fibrosis across a variety of kidney diseases 49-51. Although NLRP3 has been reported to be associated with EMT in tubular epithelial cells and murine fibroblast proliferation after TGF-β1 stimulation 52, 53, its direct involvement in regulating MMT during renal fibrosis remains to be determined. In the present study, by using NLRP3 KO mice, we uncovered the profibrotic role of NLRP3 in renal fibrosis. Because NLRP3 was predominantly expressed by macrophages, we then further explored the functional role for macrophage NLRP3 in renal fibrosis by specifically deleting NLRP3 from myeloid cells, with the novel finding that mice specifically lacking macrophage NLRP3 were protected against UUO-induced and IRI-induced progressive renal fibrosis. Thus, NLRP3 is profibrotic and functions to play a pathogenic role in renal fibrosis, which is a novel and significant finding from this study.

We also uncovered the novel mechanism that macrophage NLRP3 mediates MMT and renal fibrosis by directly interacting with TGF-β1 receptors to trigger the activation of downstream TGF-β/Smad3 signaling. In the canonical TGF-β signaling pathway, binding of TGF-β to the extracellular domain of TGF-β receptor II induces its intracellular domain to interact with and activate the intracellular domain of TGF-β receptor I, leading to downstream Smad protein activation 39. Consequently, the intracellular domains of TGF-β receptor I and TGF-β receptor II are essential for signal transduction. In the present study, we demonstrated that NLRP3 binds to the intracellular domains of both TGF-β receptor I and TGF-β receptor II and promotes their interaction. These findings suggest that NLRP3 enhances TGF-β signaling by facilitating TGF-β receptor I-TGF-β receptor II intracellular domain association, thereby amplifying the downstream Smad activation and contributing to renal fibrosis. It has been reported that macrophages mediate renal fibrosis via the process of MMT in both human and mice 14. It is also well established that activation of Smad3 signaling plays a key role in MMT and renal fibrosis via the Smad3-Src/Pou4f1 pathway 16, 17, 26. Thus, the identification of the NLRP3/Smad3/MMT axis may be a new pathway through which NLRP3 mediates MMT and renal fibrosis.

Targeting NLRP3 inflammasome activation has been shown as a potential therapeutic strategy in CKD 54. It has been reported that MCC950, a specific inhibitor of NLRP3 activation, can suppress kidney injury and fibrosis in diabetic nephropathy 55, lupus nephritis 56 and crystal nephropathy 57. However, its hepatotoxicity may limit its clinical application 58. Other NLRP3 inhibitors have also been reported to have beneficial effect on several renal disease models, while these are limited to preclinical studies 45, 59-62. OLT1177 (Dapansutrile), an active β-sulfonyl nitrile compound that inhibits NLRP3 ATPase activity and blocks NLRP3 inflammasome activation, has advanced to phase II clinical trials and demonstrated favorable efficacy in treating gouty arthritis 63, but its role in renal fibrosis remains to be investigated. In the present study, we found that the use of a new NLRP3 specific inhibitor (LicoB), which functions to disrupt the interaction between NEK7 and NLRP3, could significantly inhibit the activation of NLRP3 inflammasome in macrophages by disrupting the interaction between NLRP3 and TGF-β receptor II. This resulted in inhibiting the profibrotic function of NLRP3 by decreasing the expression of TGF-β receptor II and p-Smad3 in macrophages, thereby protecting against TGF-β1, UUO and IRI-induced MMT and renal fibrosis *in vitro* and *in vivo*. Taken together, LicoB represents a potential therapeutic candidate for renal fibrosis, whereas further studies are needed to evaluate its safety, bioavailability, and therapeutic efficacy in preclinical and clinical settings before translation into clinical practice.

In conclusion, NLRP3 is largely expressed by inflammatory macrophages and functions to play a profibrotic role in renal fibrosis. Macrophage NLRP3 mediates renal fibrosis by triggering TGF-β/Smad3-mediated MMT. Thus, targeting NLRP3 may be a novel therapy for renal fibrosis.

## Supplementary Material

Supplementary materials and methods, figures and tables.

## Data Availability

The data that support the findings of this study are openly available in repositories as described in “Cell clustering of single-cell RNA-seq data”.
