# Supplementary material for: Bone Marrow-Derived Macrophage NLRP3 Mediates Renal Fibrosis by triggering TGF-β/Smad3-mediated Macrophage-Myofibroblast Transition
Source: Int J Biol Sci. 2026 Apr 23;22(9):4705–23. doi: 10.7150/ijbs.131452 (PMC13182244; doi:10.7150/ijbs.131452)
Supplement: Supplementary file 1 — Supplementary materials and methods, figures and tables. [file ijbsv22p4705s1.pdf]

Bone Marrow-Derived Macrophage NLRP3 Mediates Renal Fibrosis by triggering TGF-β/Smad3-mediated Macrophage-Myofibroblast Transition

Supplementary Information

Supplementary Materials and Methods

Reagents

Lipopolysaccharide (LPS), phorbol-12-myristate-13-acetate (TPA), Ac-YVAD-cmk, adenosine triphosphate (ATP) and Nigericin were obtained from InvivoGene Biotech Co., Ltd. (San Diego, CA, USA). MG132 (S2619) and chloroquine (S6999) were obtained from Selleck Chemicals. RPMI 1640 and Dulbecco modified Eagle medium (DMEM) were obtained from Gibco (Grand Island, NY, USA). Antibody against Flag (F3165), HA (H6908) and monoclonal mouse anti-GAPDH (G9295) were purchased from Sigma (St. Louis, MO, USA). Monoclonal rabbit anti-NLRP3 (D2P5E), monoclonal rabbit anti-IL-1β (D3U3E) and monoclonal rabbit anti-Caspase-1 (D7F10) were purchased from Cell Signaling Technology (Beverly, MA, USA). Monoclonal mouse anti-ASC (sc-271054) and monoclonal mouse anti-TGF-β receptor II(SC-17792) were purchased from Santa Cruz Biotechnology (Santa Cruz, CA, USA). Monoclonal mouse anti-NLRP3 (AG-20B-0014-C100) was purchased from Adipogen to detection endogenous NLRP3 in THP1 cells and primary BMDMs. monoclonal rabbit anti-TGF-β receptor I(ab288303), polyclonal rabbit anti-α-SMA (ab5694), monoclonal rabbit anti-Smad3 (phospho S423 + S425) (ab52903) and polyclonal rabbit anti-Smad3(ab28379) were purchased from Abcam (Waltham, MA, USA). TGF-β1 was purchased from PeproTech (Cranbury,NJ,USA). Lipofectamine 2000, normal rabbit IgG and normal mouse IgG were purchased from Invitrogen Corporation (Carlsbad, CA, USA).

Measurement of Serum Lactate dehydrogenase (LDH), Alanine aminotransferase (ALT) and Aspartate aminotransferase (AST) levels

Aspartate aminotransferase Assay Kit and Alanine aminotransferase Assay Kit

(Najing Jiancheng Bioengineering Insitutue) were used for AST and ALT detection, and Lactate dehydrogenase assay kit (Najing Jiancheng Bioengineering Insitutue) used for LDH detection according to the manufacturer's protocol.

Opal Multiplex Immunofluorescence

For Opal multiplex immunofluorescence, sections of paraffin kidney section (3μm) from mice using a microwave-based antigen retrieval technique. After incubation with primary antibody as follows: F4/80 (Bio-rad MCA497) and a-SMA (Abcam 230458) 4°C overnight, the sections were washed using distal water and incubated with EnVision+ System-HRP Labelled Polymer Anti-Rabbit (DAKOK4003) at room temperature for 1h, then the fluorescence was developed using the Alex Fluor TM 488 Tyramide Reagent(invitrogenB40953) or Alex Fluor TM 555 Tyramide Reagent (invitrogenB40955) according to the manufacturer's protocol. The nuclei were counterstained with Hoechst 33342, Trihydrochloride, Trihydrate (invitrogen). All slides were mounted with medium and then analyzed with Leica CRT6000 fluorescence microscope.

Nuclear and Cytoplasmic Extraction

The Thermo Scientific NE-PER Nuclear and Cytoplasmic Extraction Kit (Invitrogen, 78835) was provided for efficient cell lysis and extraction of separate cytoplasmic and nuclear protein fractions and then analyzed by Western blot using target antibody.

Western Blot Analysis

HEK293T whole-cell lysates were prepared by lysing cells with buffer (50 mM Tris-HCl, pH7.5, 300 mM NaCl, 1% Triton-X, 5 mM EDTA and 10% glycerol). The TPA-differentiated THP-1 cells lysates were prepared by lysing cells with buffer (50 mM Tris-HCl, pH7.5, 150 mM NaCl, 0.1% Nonidetp 40, 5 mM EDTA and 10% glycerol). Protein concentration was determined by Bradford assay (Bio-Rad, Hercules, CA, USA). Cultured cell lysates (30 μg) were electrophoresed in an 8-12% SDS-PAGE gel and transferred to a PVDF membrane (Millipore, MA, USA). PVDF membranes were blocked with 5% skim milk in phosphate buffered saline with 0.1% Tween 20

(PBST) before being incubated with the antibody. Protein band were detected using a Luminescent image Analyzer (Tanon,4600SF).

Plasmid construction

The cDNAs encoding human NLRP3, TGF-β receptor I, TGF-β receptor II, Smad2, Smad3 and Smad4 were obtained by reverse transcription of total RNA from TPA-differentiated THP-1 cells, followed by PCR using specific primers. The cDNAs were sub-cloned into pcDNA3.1(+) and pcagg-HA vector. The pcDNA3.1(+)-3×Flag vector was constructed from pcDNA3.1(+) vector through inserting the 3×Flag sequence between the NheI and HindIII site. Following are the primers used in this study.

Flag-NLRP3:
5'-CGCGGATCCATGAAGATGGCAAGCACCCGC-3',
5'-CCGCTCGAGCTACCAAGAAGGCTCAAAGAC-3';
HA-TGF-β receptor I:
5'-CATCATTTTGGCAAAGAATTCATGGAGGCGGCGGTCGCT-3',
5'-TGCATCGATGAGCTCGAATTCTTACATTTTGATGCCTTCCTGTTG-3';
HA- TGF-β receptor II:
5'-TGCATCGATGAGCTCGAATTCCTATTTGGTAGTGTTTAGGGAGCCG-3',
5'-TGCATCGATGAGCTCGAATTCCTATTTGGTAGTGTTTAGGGAGCCG-3';
HA-SMAD2:
5'-CATCATTTTGGCAAAGAATTCATGTCGTCCATCTTGCCATTCA-3',
5'-TGCATCGATGAGCTCGAATTCTGACATGCTTGAGCAACGCA-3';
HA-SMAD3:
5'-CATCATTTTGGCAAAGAATTCATGTCGTCCATCCTGCCTTTC-3',
5'-TGCATCGATGAGCTCGAATTCCTAAGACACACTGGAACAGCGG-3';
HA-SMAD4:
5'-CATCATTTTGGCAAAGAATTCATGGACAATATGTCTATTACGAATACACC-3',5'-TGCATCGATGAGCTCGAATTCGTCTAAAGGTTGTGGGTCTGCAA-3';
HA- TGF-β receptor I D1:
5'-CATCATTTTGGCAAAGAATTCATGGAAGCCGCTGTGGCC-3',
5'-TTGGCAGAGGGAAAAAGATCTTCAAGCGTAGTCTGGGACGTC-3';
HA- TGF-β receptor I D2:
5'-CATCATTTTGGCAAAGAATTCTGCCACAACAGAACCGTGATC-3',
5'-TTGGCAGAGGGAAAAAGATCTTCAAGCGTAGTCTGGGACGTC-3';
HA- TGF-β receptor II D1:
5'-CATCATTTTGGCAAAGAATTCATGGGTAGAGGTCTGCTTCGC-3',
5'-TTGGCAGAGGGAAAAAGATCTTCAAGCGTAGTCTGGGACGTC-3';
HA- TGF-β receptor II D2:
5'-CATCATTTTGGCAAAGAATTCTGCTATCGGGTGAACCGGC-3',
5'-TTGGCAGAGGGAAAAAGATCTTCAAGCGTAGTCTGGGACGTC-3'.

The PYRIN, NACHT, and LRR domain of NLRP3 protein was cloned into pcDNA3.1(+). The TGF-β receptor I and TGF-β receptor II truncates was cloned into pcaggs-HA vector using specific primers, which are listed as follows.

Flag-PYRIN:

5'-AAAGGATCCATGAAGATGGCAAGCACCCGC-3',

5'-CGGCTCGAGCTATAAACCCATCCACTCCTCTTC-3';

Flag-NACHT:

5'-AAAGGATCCCTGGAGTACCTTTCGAGAATCTC-3',

5'-CCCCTCGAGCTAGATCTTGCAACTTAATTTCTTC-3';

Flag-LRR:

5'-AAAGGATCCTCTCAGCAAATCAGGCTGGAG-3',

5'-CGGCTCGAGCTACCAAGAAGGCTCAAAGACG-3'.

Real-Time PCR

Total RNA was extracted with TRIzol reagent (Invitrogen), following the manufacturer's instructions. Real-time quantitative-PCR was performed using the Bio-Rad CFX96 Touch and SYBR qRT-PCR kits (Bio-Rad) in a reaction mixture of 20 μl SYBR Green PCR master mix, 1 μl DNA diluted template, and RNase-free water to complete the 20 μl volume. Real-time PCR primers were designed by Primer Premier 5.0 and their sequences were as follows:

Mouse collagen I forward, 5'- TCCTGGCAACAAAGGAGACA-3,

Mouse collagen I reverse, 5'- GGGCTCCTCGTTTTCCTTCT-3',

Mouse a-SMA forward, 5'- CCCAGACATCAGGGAGTAATGG-3',

Mouse a-SMA reverse, 5'- TCTATCGGATACTTCAGCGTCA-3',

Mouse GAPDH forward, 5'- GGTGAAGGTCGGTGTGAACG-3',

Mouse GAPDH reverse, 5'- CTCGCTCCTGGAAGATGGTG-3'.

Co-Immunoprecipitation Assays

HEK293T whole-cell lysates were prepared by lysing cells with buffer (50 mM

Tris-HCl, pH7.5, 300 mM NaCl, 1% Triton-X, 5 mM EDTA, and 10% glycerol). TPA-differentiated THP-1 cells lysates were prepared by lysing cells with buffer (50 mM Tris-HCl, pH7.5, 150 mM NaCl, 0.1% Nonidetp40, 5 mM EDTA, and 10% glycerol). Lysates were immunoprecipitated with control mouse immunoglobulin G (IgG) (Invitrogen) or anti-Flag antibody (Sigma, F3165) with Protein-G Sepharose (GE Healthcare, Milwaukee, WI, USA).

Supplementary Legends

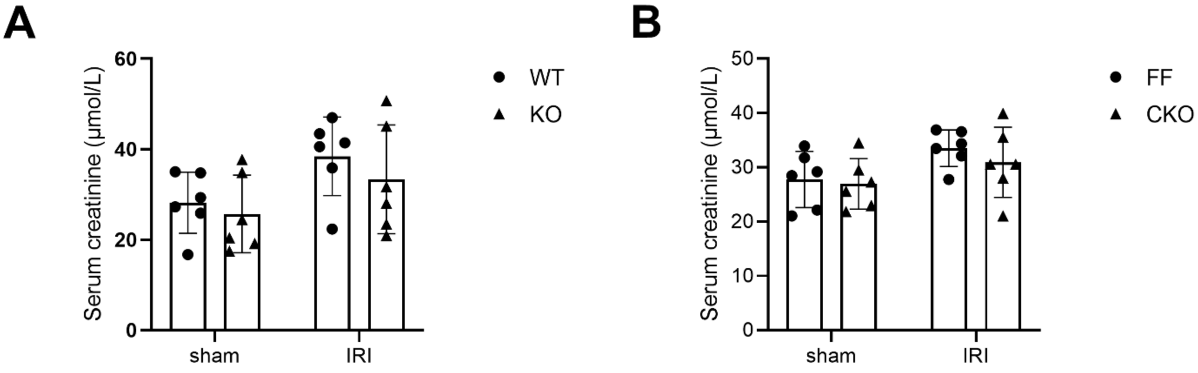

Figure S1. Serum creatinine of IRI-induced renal fibrosis mice. (A-B) Serum creatinine levels were measured in NLRP3 KO (A) and NLRP3 cKO (B) mice at 14 days after IRI.

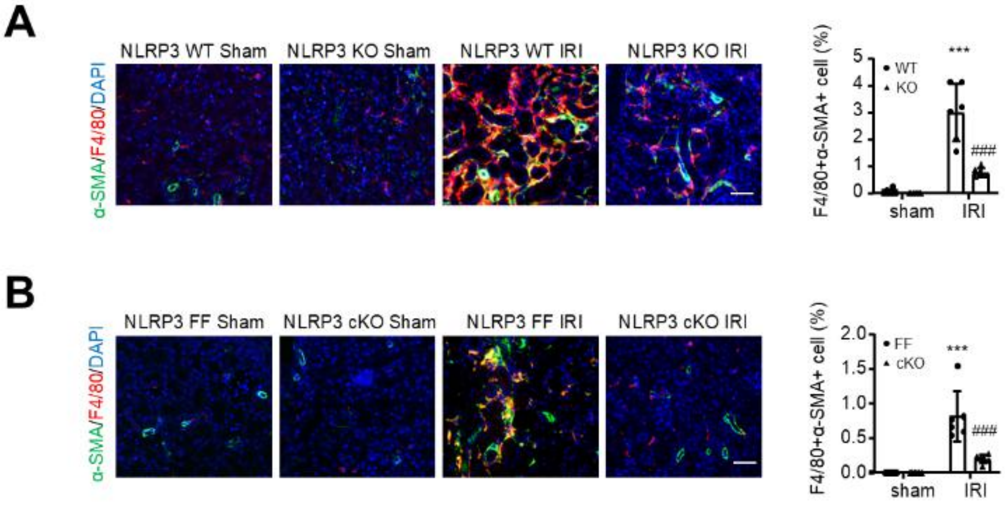

Figure S2. MMT (a-SMA+CD68+) cells were detected by two-color immunofluorescence in the fibrosing kidney. Tow-color immunofluorescence

identify the expression of MMT cells (α-SMA, green; F4/80, red) in NLRP3 KO(A) and NLRP3 CKO(B) on IRI-induced renal fibrosis. Each dot represents one mouse and data are expressed as the mean ± SEM for groups of six mice. ***p < 0.001 versus NLRP3 WT Sham, NLRP3 Flox/Flox Sham or NLRP3 WT Mock; ###p < 0.001 versus NLRP3 WT IRI or NLRP3 Flox/Flox IRI. Scale bars = 50 μm.

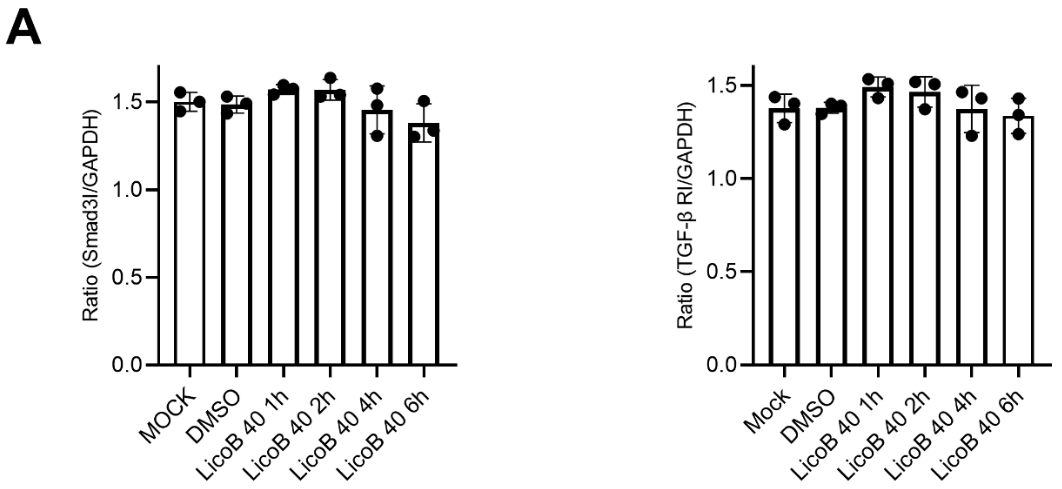

Figure S3. Quantification of TGF-β receptor I and Smad3 protein levels shown in Figure 9C.

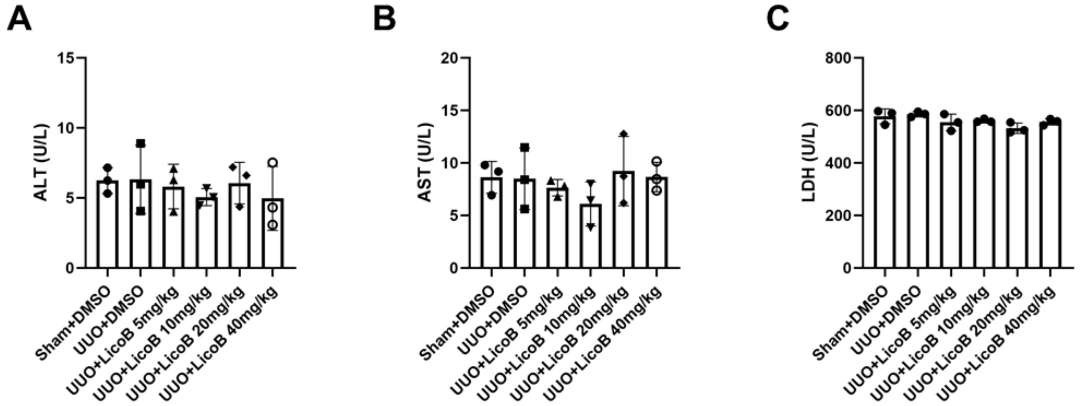

Figure S4. The toxicity of LicoB different dose in C57BL/6 mice in each group. (A) Alanine aminotransferase (ALT) levels in each group.(B) Aspartate aminotransferase (AST) levels in each group.(C) Lactate dehydrogenase (LDH) levels in each group. Each bar represents the mean ± SEM for groups of 3 mice. Student's t test and one-way ANOVAs are used for comparison between two groups and multiple groups,

respectively.

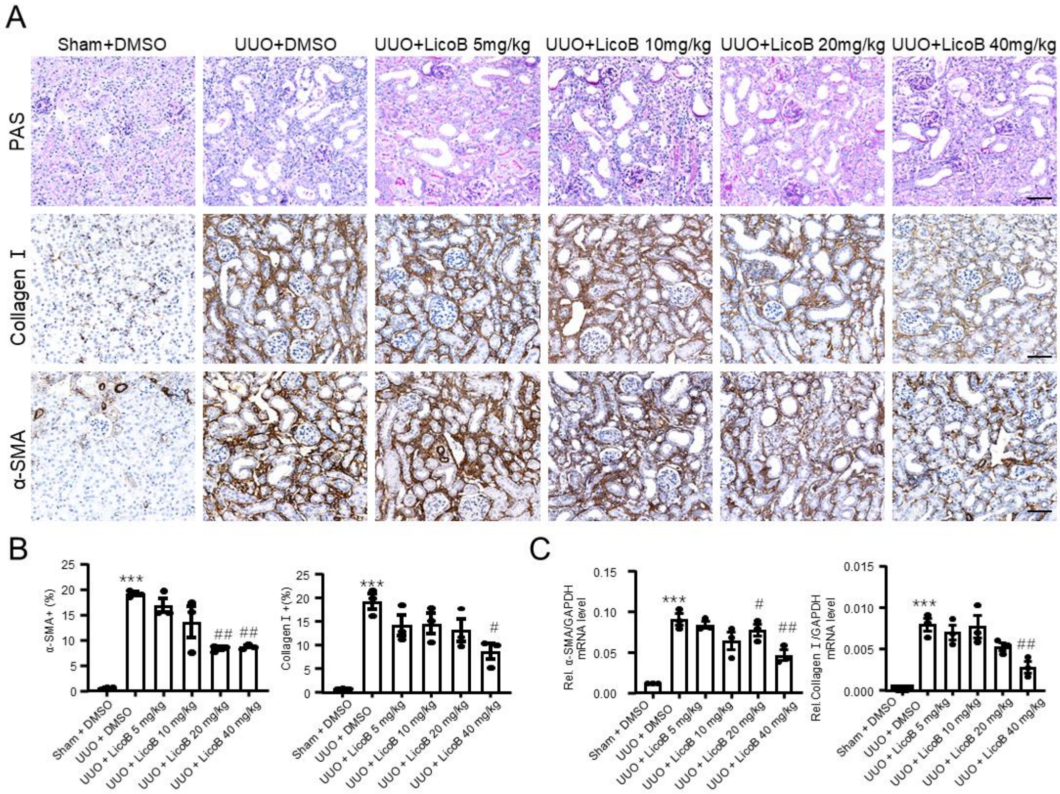

Figure S5. Treatment with a NLRP3 inhibitor blocks UUO-mediated renal fibrosis. Licochalcone B at a dose of 5,10,20 and 40 mg/kg/day was treated in UUO. (A and B) PAS staining and Immunohistochemistry for detecting collagen I and α-SMA on day7 UUO(A). Semiquantitative analysis of positive area in each group(B). (C) Real-time PCR for levels of collagen I and α-SMA. Each bar represents the mean ± SEM for groups of 3 mice. ***$p < 0.001$ versus Sham+DMSO; #$p < 0.05$, ##$p < 0.01$ versus UUO+DMSO. Scale bars = 50 μm.

Table S1. The number of NLRP3 positive cells in each cluster.

	NLRP3-	NLRP3+
T cell	474	10
NK	733	7
B cell	3265	3
Monocyte	2061	969
Macrophage	856	255
DC	586	255
Basophils	129	13
Myo-/fibroblast	2067	3
Pericyte	638	5
vSMC	426	0
Endothelial	29749	65
Podocyte	44	0
PT	1820	6
LOH	3067	5
CNT	2936	7
IC	1389	6
